# Medical and Philosophical News

**Published:** 1781-06

**Authors:** 


Mr. James Kerr, ſurgeon in Bengal, to whom the public are already indebted for a deſcription of of the tree producing the *terra japonica*, inferted in the Med. Obf. & Inq. Vol. V. has lately tranfmitted to the Royal Society a curious account of the infect that produces gum lacca.

